# The impact of formative testing on study behaviour and study performance of (bio)medical students: a smartphone application intervention study

**DOI:** 10.1186/s12909-015-0351-0

**Published:** 2015-04-10

**Authors:** Anke L Lameris, Joost GJ Hoenderop, René JM Bindels, Thijs MH Eijsvogels

**Affiliations:** 1 Radboud university medical center, Radboud Institute for Molecular Life Sciences, Nijmegen, Netherlands; 2 Department of Physiology, Radboud university medical center, Radboud Institute for Health Sciences, PO box 9101, 6500 HB Nijmegen, The Netherlands

**Keywords:** Formative testing, E-learning, Medical education, Blended learning, App

## Abstract

**Background:**

Formative testing can increase knowledge retention but students often underuse available opportunities. Applying modern technology to make the formative tests more attractive for students could enhance the implementation of formative testing as a learning tool. This study aimed to determine whether formative testing using an internet-based application (“app”) can positively affect study behaviour as well as study performance of (bio)medical students.

**Methods:**

A formative testing app “Physiomics, to the next level” was introduced during a 4-week course to a large cohort (n = 461) of Dutch first year (bio)medical students of the Radboud University. The app invited students to complete 7 formative tests throughout the course. Each module was available for 3-4 days to stimulate the students to distribute their study activities throughout the 4-week course.

**Results:**

72% of the students used the app during the course. Study time significantly increased in intensive users (p < 0.001), while no changes were observed in moderate (p = 0.07) and non-users (p = 0.25). App-users obtained significantly higher grades during the final exam of the course (p < 0.05). Non-users more frequently failed their final exam (34%, OR 3.6, 95% CI: 2.0-6.4) compared to moderate users (19%) and intensive users (12%). Students with an average grade <6.5 during previous courses benefitted most from the app, as intensive (5.8 ± 0.9 / 36%) and moderate users (5.8 ± 0.9 / 33%) obtained higher grades and failed their exam less frequently compared to non-users (5.2 ± 1.1 / 61%). The app was also well appreciated by students; students scored the app with a grade of 7.3 ± 1.0 out of 10 and 59% of the students indicated that they would like the app to be implemented in future courses.

**Conclusions:**

A smartphone-based application of formative testing is an effective and attractive intervention to stimulate study behaviour and improve study performance in (bio) medical students.

## Background

As a result of a recently implemented legally binding study advice, Dutch students have to perform well during their education. To remain in the study program students are required to obtain at least 40 out of a total of 60 credits during the propaedeutic phase. Although study performance in the 1^st^ year of the study program is essential, we frequently observed that many (bio) medical students start late with their preparations for the examinations.

Repeated study sessions are beneficial for knowledge retention [1,2]. The method of test enhanced learning, also known as formative testing/assessment, uses frequent tests as a learning tool to increase the retention of information. Formative testing stimulates learning processes and knowledge retention in both direct and indirect ways [3]. Direct effects of testing include improved retention and understanding, resulting from the act of successfully retrieving information from memory. Indirect effects of testing refer a broad range of other ways in which testing can influence learning [3]. The idea that testing only assesses the content of memory is outdated: learning actually occurs during the process of testing, which stimulates long-term knowledge retention [4,5]. It has been suggested that this is merely the result of increased exposure to the study material during testing [6,7]. However, several studies have shown that taking test results in better knowledge retention than re-studying the material for an equal amount of time [8,9]. The retrieval of information from memory during testing strengthens the memory regarding that information leading to increased long-term retention [10,11]. In addition to these direct effects, formative testing stimulates the students to spread their study activities over time and allows students to identify their areas of weakness after which they can study the related material more purposefully [3].

Although there is a large body of evidence showing that test-enhanced learning effectively increases knowledge retention, recent studies indicate that students often underuse available formative testing opportunities [12,13]. E-learning methods have been shown to be effective and well-appreciated teaching tools in a large variety of (bio) medical educational settings [14-17]. Applying modern technology to make the formative tests more attractive for students could help in the implementation of formative testing as a learning tool for (bio) medical teaching. In this study, an internet-based application, or “app”, called “Physiomics, to the next level” was introduced during a 4-week course for first year (bio) medical students.

The aim of this study was to determine whether the use of an internet-based application with a formative testing approach can stimulate study behavior as well as study performance in a large cohort of first year (bio) medical students. We hypothesized that students who use the app will spend more time studying during the first weeks of the course. In addition, we anticipate that students who use the app perform better during the final exam.

## Methods

### Population

336 medicine students and 125 biomedical sciences students who registered for the course “Circulation and Respiration” received an invitation to participate in this study. Before using the app, students were informed about the study and informed consent was obtained. The study was approved by the educational advisory board of the Radboud university medical center, and we adhered to the Declaration of Helsinki during the study design, data collection and data analysis.

### Study and app design

The “Physiomics to the next level” app was designed as an open-source HTML-based application and could be used on all major operating systems and devices, including cell phones, tablets, desktops and laptops. A Dutch demonstration version of the app can be accessed via a guest account at www.physiomics.eu/app.

Students were invited to use the app via email, through the virtual learning environment Blackboard, and during the first lecture of the course. During the first day of the 4-week course, students received an email with a personal password connected to their email address that allowed them access to the app. In the app, students had access to a tutorial course, in which they could familiarize themselves with the use of the app, as well as to a course specific section. Matching the structure of the course, the app was subdivided into 7 subsequent modules covering different topics. Each module consisted of 10 multiple-choice questions. When the students answered ≥7 questions correctly, 5 additional bonus questions were unlocked. Each question needed to be answered within 60 seconds and questions could only be answered once. Completed questions remained available for review purposes at later time points. As an incentive, students were informed that out of the total number of 35 bonus questions, 5 questions would reappear on their final exam. The bonus questions of each module could only be unlocked during a 3-4 day time frame, stimulating the students to spread the study-load evenly throughout the 4-week course. Feedback regarding the answers to the questions in the app was provided directly by means of a green checkmark or a red cross. In case a wrong answer was given, a pop-up appeared referring the student to relevant pages in their course-guide and textbook where they could search for the right answer. At the end of the 4-week “Circulation and Respiration” course, assessment took place via a written examination consisting of multiple-choice questions. Grades for the final exam can vary between 0 (lowest score) and 10 (highest score), students pass an exam when they obtain a grade ≥5.5.

### Data-collection

A number of parameters was logged during the use of the app, including the answer given to each question, the time spent on answering a question and the number of questions answered correctly. After the final exam, examination grades were collected. In addition, detailed information regarding the individual scores for the included bonus questions was obtained. After the final exam students were requested to fill in a questionnaire concerning the use of the app and their study behavior. The Student Identification Number was used to merge the data from the app, examination score and questionnaire. In contrast to its name, this seven-digit number cannot be linked to personal identifiers by third party’s other than the Administration Department of the Radboud University. Therefore, anonymity of data was guaranteed in our study.

In the questionnaire, students were asked to provide an estimation of the number of hours they spent studying per week for each individual week of a 4-week course (multiple choice: 0-10 hours, 11-20 hours, 21-30 hours, 31-40 hours, 51-50 hours, >60 hours). They were asked to provide this information for the “Circulation and Respiration” course as well as the average study time during previous courses which were similar in duration and set-up. These data were used as a parameter to measure study behavior. Study performance was assed based on the grade obtained during the final examination of the “Circulation and Respiration” course. To correct for previous performance, grades of the same student cohort during 4 previous courses were collected as well, and matched to the dataset.

### Statistical methods

Statistical analyses were performed using the Statistical Package for the Social Sciences (IBM SPSS Statistics for Mac, Version 20.0. Armonk, NY: IBM Corp.). A dummy variable was introduced to classify a student into a “non-user” (completed 0 modules), “moderate user” (completed 1-4 modules) or “intensive user” (completed 5-7 modules). Similarly, students were classified as “below average” (0- < 6.5), “average” (6.5-7.5) or “above average” (>7.5-10) based on their average historical grade, calculated from the final examination grades obtained during 4 previous courses. Quantitative data is presented as means ± standard deviation (SD), categorical variables are presented by percentage. Differences in study behaviour (dependent variable) between the current and previous courses across the 4 weeks of teaching were assessed using a two-way repeated measures analysis of variance with Greenhouse-Geisser corrections. Differences in examination grade, bonus question score and corrected examination score were compared between user groups using a one-way analysis of variance with LSD post-hoc test The effect of the app on the risk to fail for the final exam was analyzed using binary logistic regression analysis. Odds Ratios are presented with 95% confidence intervals (CI). Results were considered significant in the situation that p < 0.05.

## Results

### Participants

In total, 461 students were invited to use the app. Students that did not take the final exam or did not enroll in the course were excluded from the data analysis (n = 14). In addition, students of which no previous grades were available (n = 8) were excluded. This resulted in a final study population of 439 students. The majority of the students was female (66% female, 34% male) and studied medicine (74% medicine, 26% biomedical sciences) (Table 1). The percentage female students in the non-user, moderate user and intensive user groups was significantly different (p < 0.001). No differences were observed in the ratio of medicine and biomedical sciences students between groups (p = 0.38). In total, 72 % of the students used the app.

**Table 1:** **Group characteristics of the study cohort**

	Total study population	Non-users	Moderate users	Intensive users	*p* value
Number of students	439	122	139	178	
Sex					<0.001
Female (%)	66	50	69	74	
Male (%)	34	50	31	26	
Study					0.38
Biomedical Sciences (%)	26	21	29	26	
Medicine (%)	74	79	71	74	

### Study behavior

The number of hours students spent on studying increased gradually during a 4-week course for non-users as well as moderate and intensive users (Table 2). For non-users, study hours were similar during the current course (in which the app was implemented) compared to previous courses (p = 0.25). Moderate users of the app tend to spend more time studying during the current course compared to previous courses (p = 0.07). In the intensive user group the difference between study-time in the current course compared to previous courses was most pronounced (p < 0.001).

**Table 2:** **Study behaviour throughout the 4-week course**

		Week				Repeated measurements ANOVA		
		1	2	3	4	Week	Course	Interaction
Study hours score of non-users						**p < 0.001**	p = 0.265	p = 0.246
	Previous courses	2.4 ± 1.1	2.7 ± 1.1	3.2 ± 1.1	4.1 ± 1.3			
	Circulation & Respiration	2.1 ± 1.1	2.8 ± 1.2	3.2 ± 1.3	4.1 ± 1.4			
Study hours score of moderate users						**p < 0.001**	p = 0.161	**p = 0.070**
	Previous courses	2.7 ± 1.1	2.9 ± 1.1	3.4 ± 1.2	4.2 ± 1.3			
	Circulation & Respiration	2.7 ± 1.1	3.0 ± 1.1	3.5 ± 1.2	4.4 ± 1.2			
Study hours score of intensive users						**p < 0.001**	**p = 0.016**	**p < 0.001**
	Previous courses	3.2 ± 1.1	3.4 ± 0.9	3.7 ± 0.9	4.4 ± 1.1			
	Circulation & Respiration	3.1 ± 1.1	3.6 ± 1.1	4.0 ± 1.3	4.5 ± 1.1			

Number of hours students spent studying per week for each individual week of a 4-week course (1 = 0-10 hours, 2 = 11-20 hours, 3 = 21-30 hours, 4 = 31-40 hours, 5 = 51-50 hours, 6= > 60 hours).

### Study performance

**Figure 1:**
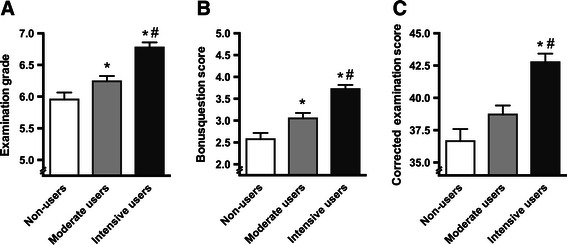
**Grades and scores on final examination. A**. Grades on the final exam subdivided by non-users (white bars), moderate users (grey bars) and intensive users (black bars). **B**. Score on bonus questions in the final exam. **C**. Corrected examination scores (total score minus bonus question score). Data are presented as means ± SEM. *p < 0.05 compared to non-users, ^#^p < 0.05 compared to moderate users.

On average, students scored a 6.4 ± 1.1 on the final exam of the course. Both moderate (p = 0.036) and intensive users (p < 0.001) scored significantly higher on the final exam compared to non-users and compared to each other (p < 0.001) (Figure 1A). Both moderate (p = 0.007) and intensive users (p < 0.001) scored significantly better on the 5 bonus questions in the final exam compared to the non-users as well as compared to each other (p < 0.001) (Figure 1B). When corrected for the score on these bonus questions, intensive users of the app scored significantly better on the final exam compared to non-users (p < 0.001) as well as moderate users (p < 0.001), moderate users also tended to score better than non-users (p = 0.07) (Figure 1C).

In total, 22% of all students failed to pass their final exam. The percentage of students failing for the exam correlates with the use of the app. Non-users more frequently failed their exam (34%) compared to moderate users (19%; OR 0.46, CI 0.26-0.80) and intensive users (12%; OR 0.28, CI 0.16-0.50).

### Subgroup analysis

Based on the average grade during 4 previous 4-week courses, students were classified as “below average” (n = 162, 37%), “average” (n = 155, 35%), or “above average” (n = 122, 28%) (Table 3). “Above average” students had a lower chance (OR 0.4, CI 0.2–0.7) to be in the non-user group, but higher chance (OR 3.2, CI 1.9-5.2) to be in the intensive-user group, compared to the “below average” group. Likewise, “average” students had a higher chance (OR 1.9, CI 1.2-3.1) to be in the intensive-user group compared to the “below-average” group. Therefore, students were divided into subgroups of non-users, moderate users and intensive users across the historical grade groups.

**Table 3:** **Subgroup characteristics based on historical grades**

	Total study population	“Below average”				“Average”				“Above average”			
		NU	MU	IU	p value	NU	MU	IU	p value	NU	MU	IU	p value
Number of students (n)	439	56	61	45		45	44	66		21	34	67	
Sex					0.006				0.030				0.22
Female (%)	66	43	62	73		53	68	77		62	82	70	
Male (%)	34	57	38	27		47	62	23		38	18	30	
Study					0.42				0.18				0.17
Biomedical Sciences (%)	26	34	41	47		13	30	23		5	6	16	
Medicine (%)	74	66	59	53		87	70	77		95	94	83	

NU: non users, MU: moderate users, IU: intensive users.

**Figure 2:**
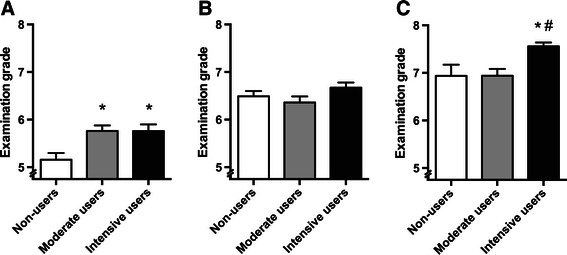
**Grades and scores corrected for previous performance.** Average grades of non-users (white bars), moderate users (grey bars) and intensive users (black bars) during on the final exam of students with “below average” **(A.)** “average” **(B.)** and “above average” **(C.)** historical grades. Data are presented as means ± SEM. *p < 0.05 compared to non-users, ^#^p < 0.05 compared to moderate users.

In the group of students classified as “below average”, use of the app resulted in a significantly higher grade during the final examination for both moderate (p = 0.001) and intensive users (p = 0.002) compared to non-users (Figure 2A). No significant differences were present between the subgroups of the “average” students (p = 0.15) (Figure 2B). In the group of students classified as “above average”, only intensive users of the app obtained a significant higher grade during the final exam (p < 0.001) (Figure 2C).

Within the “below average” historical grade group, non-users more frequently failed their exam (61%) compared to moderate users (33%; OR 0.32, CI 0.15-0.67) and intensive users (36%; OR 0.36, CI 0.16-0.81). Due to the low number of students that failed the final exam within the “average” and “above average” historical grade groups no significant differences between user groups were found.

### Evaluation

**Figure 3:**
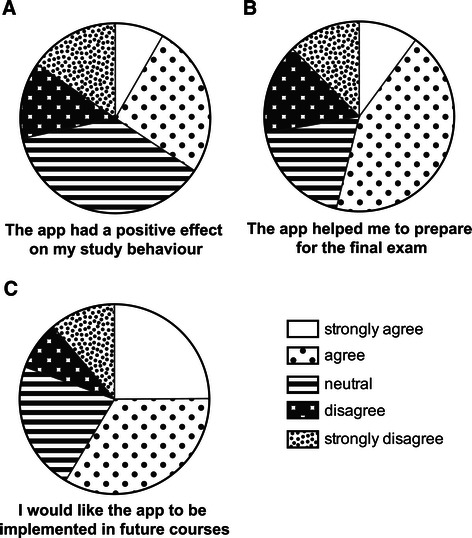
**Evaluation of the app.** Students were asked whether they (strongly) agreed, were neutral or (strongly) disagreed with a number of statements. **A**. “Using the app positively affected my study behavior”. **B**. “The app helped me to prepare for the final exam”. **C**. “I would like to see the app implemented in other courses”.

Students scored the app with an average grade of 7.3 ± 1.0. The layout and user friendliness were graded with 7.9 ± 1.1 and 7.0 ± 1.4, respectively. 34% of the respondents felt that the app positively affected their study behavior (Figure 3A), and 54% of the respondents stated the app helped them in their exam preparations (Figure 3B). Finally, a majority of the respondents (59%), indicated they would like the app to be implemented in future courses (Figure 3C).

## Discussion

The current study shows that users of an internet-based app called “Physiomics, to the next level”, which provides students with a series of formative tests, display better study behavior as well as study performance compared to non-users in a large cohort of first year (bio) medical students. These data suggest that internet-based formative testing constitutes a powerful and innovative tool for (bio) medical teachers to positively reinforce student behaviour and performance.

The “Physiomics, to the next level” app was successfully implemented during a 4-week course, as indicated by the fact that 72% of the students used the app. Other studies applying e-learning programs found similar participation grades [18-20,16]. Users of the app spent more hours studying per week compared to previous courses; this difference in study behavior was absent in non-users. These data suggest that the app positively affects study behavior. Interestingly, there was a significant baseline difference in the amount of time invested in study between the non-users, moderate users and intensive users, which could represent an important confounding factor for the examination grade as it suggests that the users of the app are in general higher achievers compared to the non-users. Indeed, previous studies have shown that students that generally perform well are more likely to use available e-learning methods [21,22].

In addition to differences in study behavior, there were also variances in study performance between the user-groups. Users of the app obtained significantly higher grades on the final exam of the course compared to non-users. According to recent studies in sixth and eight grade students, formative testing increases the percentage of correctly answered questions during summative tests by ±10% [23,24]. In a group of undergraduate students, a 14% increase in knowledge retention after 1 week was observed when students underwent formative testing [8]. In our study, the difference between the performance of non-users and intensive users on the score during the final exam was approximately 8 %. This is slightly lower compared to previous studies, however, these other studies took place under controlled circumstances in which students either repeatedly studied the material or took repeated formative tests. In our study, the formative tests were part of a course in which a blended-learning approach was used. The additional exposure via the other methods may have slightly reduced the effect of app, compared to the previously published studies that were performed under controlled circumstances. Alternatively, the use of multiple-choice questions for formative testing may have reduced our effect size. Although this type of questions reflected the final examination of the course, it is known that short answer questions (production tests) generally produce better long-term retention [25]. The effect of the app on study performance may therefore have been underestimated.

Baseline differences in study behavior between groups suggested that the students that used the app, performed better in general. Indeed, they have significantly better historical examination grades compared to the non-users. Interestingly, after correction based on historical grades, it was the group of historically “below average” students that seem to benefit most from the use of the app, obtaining higher grades and having a lower risk of failing the final exam. In the average group usage of the app did not have a significant effect on the final examination grade. The “excellent” students only benefitted significantly when using the app intensively, indicating a ceiling-effect. The app could thus be an important new tool to boost the performance of the group of students that generally perform “below average”. 66% of the students in the “below average” group used the app, indicating that the app successfully engages this group of students. In general, but especially in the light of the recently implemented Dutch legally binding study advice, this is an important finding as it indicates that the app could help those students that are at risk to be excluded from the study program.

The “Physiomics, to the next level” app was developed as a practical learning tool that can be used by medical teachers. While the combination of 1) formative testing, 2) opportunities to unlock bonus questions, and 3) temporal availability of the study modules improved study behaviour and study performance, it is not possible in the current study design to determine whether this is the result of improved knowledge retention or stimulated spaced learning sessions. More importantly, however, is the fact that the students appreciated the app and showed that they favor implementation of the app in future courses. These findings are in line with previous studies which demonstrate that students are generally highly appreciative of e-learning methods as part of a blended-learning program [26]. The app thus represents not only a useful new teaching tool but also one that is well appreciated by students.

A potential weakness of this study was the lack of a control group to allow an objective comparison of the effects of the App. However, we purposely chose to give all students access to the app, in order not to withhold study-opportunities from certain students. Future improvements to the app could include more detailed feedback. In the current setting, students were only informed whether the answer they had given was right or wrong. In case a wrong answer was given, a pop-up appeared activating the student to independently search for the right answer. Some studies however indicate that including the right answer in multiple-choice questions in the feedback is critical [27]. In addition, other studies show that detailed feedback providing some information on why a certain answer is correct has better effects than providing only the right answer, improving examination outcome with 4.6% [28]. The lack of detailed feedback in our study could, therefore, have potentially resulted in an underestimation of the effect of the app.

## Conclusions

In conclusion, users of the “Physiomics, to the next level” app spend more hours studying and obtained higher grades on the final examination. Students that generally perform “below average” benefit most from the use of the app. The majority of students used this new learning tool and it was well appreciated. This internet-based application for formative testing thus represents a powerful and innovative tool which can be used as part of a blended-learning environment to stimulate both study behavior and performance in (bio) medical teaching.
